# Parasitic plants show striking convergence in host preference across angiosperm lineages

**DOI:** 10.1093/aob/mcae180

**Published:** 2025-07-14

**Authors:** Sebastian A Hatt, Olwen M Grace, Alexandre R Zuntini, Duncan D Cameron, Chris J Thorogood

**Affiliations:** Royal Botanic Gardens, Kew, London TW9 3AE, UK; Earth and Environmental Sciences, University of Manchester, Manchester M13 9PL, UK; Royal Botanic Garden Edinburgh, Edinburgh EH3 5NZ, UK; Royal Botanic Gardens, Kew, London TW9 3AE, UK; Earth and Environmental Sciences, University of Manchester, Manchester M13 9PL, UK; Department of Biology, University of Oxford, Oxford OX1 3RB, UK; University of Oxford Botanic Garden and Arboretum, Oxford OX1 4AZ, UK

**Keywords:** Parasitic plants, convergence, Hydnoraceae, Orobanchaceae, Balanophoraceae, host specificity, host preference, host range, host diversity, haustorium, co-evolution

## Abstract

**Background and Aims:**

The host specificity of a parasite underpins its ecology, distribution, invasive potential and adaptability, yet for most parasitic plants the host ranges are poorly understood. We examine host–parasite relationships across lineages to infer how host specificity might have influenced the evolution of parasitism in plants.

**Methods:**

Host preference data for all plant holoparasite species were collected manually from literature and herbarium specimens, then analysed to investigate and visualize host diversity and specificity.

**Key Results:**

We reveal a disproportionality in host preference across host lineages: the Asteraceae contain 10 % of angiosperm diversity but are infected by 31 % of parasite species; meanwhile, monocots comprise 23 % of angiosperm diversity but are infected by only 3.2 % of parasite species of parasite species. Furthermore, we observe striking convergence in host preference: Asteraceae, Euphorbiaceae and Fabaceae are infected by six, five and four independent parasite lineages, respectively. We also demonstrate considerable variation in the degree of host specificity among closely related parasite species; a result that does not reflect the expectation of holoparasites (especially endoparasites) as host specialists.

**Conclusions:**

The marked pattern of convergence in preference across disparate lineages points to a common pathway in the evolution of parasitism of eudicots in preference to monocots, which might, in turn, have been driven by a divergence in host root and vascular architecture. The unexpected variation in host specificity among closely related species suggests that even apparent generalists might contain cryptic host-specific taxa. This highlights the value of host preference as an additional consideration in parasitic plant taxonomy. Together, our data point to a complex interplay between ecological and physiological factors driving the evolution of host–parasite interactions. Moreover, they emphasize how little is known about the ecology of most holoparasitic plants, a group of organisms that are especially vulnerable at a time of unprecedented biodiversity loss and extinction.

## INTRODUCTION

Host–parasite systems are widespread across the tree of life and serve as ideal models for understanding co-evolutionary dynamics (Penczykowski *et al.*, 2016). In the plant kingdom, parasitic plants (those parasitic on other plants) comprise a diverse group of ~4750 species, representing 1.6 % of angiosperms (Nickrent, 2020). Parasitism has evolved independently ≥12 times across the angiosperm phylogeny (Nickrent, 2020). All parasitic plants possess a haustorium, which is a highly specialized organ that serves as a conduit between the host and parasite vascular tissues (Teixeira-Costa, 2021). Several species (e.g. *Striga hermonthica* and *Orobanche crenata*) are considered economically important pests responsible for significant harm to agricultural crops and forestry operations worldwide (Press and Phoenix, 2005). Conversely, many parasitic plants are described as keystone species that exert a profound influence on the surrounding ecosystem, such as *Rhinanthus*, which affects community diversity by repressing the dominance of grasses (Těšitel *et al.*, 2021).

Parasitic plants show considerable functional diversity, ranging from primarily autotrophic facultative hemiparasites to obligate holoparasites that have lost all photosynthetic ability; the most extreme examples, the endoparasites, spend their entire life cycle embedded within the tissues of their hosts, emerging only to flower and set seed (Thorogood *et al.*, 2021). Acknowledging the ongoing debate over the preferred classification of parasitic plant functional diversity (Musselman and Press, 1995; Heide-Jørgensen, 2008; Těšitel, 2016; Teixeira-Costa and Davis, 2021), we follow Těšitel (2016) in defining a holoparasite as a plant devoid of chlorophyll and thus entirely dependent on its host over its entire life cycle, including both endoparasites and other obligate root parasites, but excluding euphytoid parasites, mistletoes and parasitic vines *sensu*Teixeira-Costa and Davis (2021). Here, we focus exclusively on holoparasites because all are obligate parasites.

Host specificity defines the range and diversity of host species that a parasite is capable of infecting (Wells and Clark, 2019). Preference can exist for a particular host species, genus, family or even multiple families. Host specificity is influenced by multilayered interactions at the molecular, physiological and ecological levels (Cameron and Seel, 2007; Thorogood and Hiscock, 2010). For example, there are many compatibility checkpoints to be passed for successful parasitism of a host, including molecular recognition at germination, penetration, establishment and persistence of the parasite tissue within the host (Casadesús and Munné-Bosch, 2021).

Host specificity can alter parasite ecology, for example by influencing abundance and distribution, invasiveness potential, extinction risk, and the dynamics of plant communities more broadly. Host–parasite systems have been explored across the tree of life (Frankel *et al.*, 2015; Stokke *et al.*, 2018; Acevedo *et al.*, 2019; Kruitwagen *et al.*, 2022), but host preference in plant parasites remains relatively under-examined. Not only does this hamper our ability to conserve these poorly known plants, 76 % of which have never been cultivated or conserved anywhere (Thorogood *et al.*, 2022), but also it severely restricts our capacity to understand their taxonomy, evolution and ecosystem functioning. Here, we examine all documented host–parasite interactions among holoparasitic lineages. We identify patterns in host diversity and specificity and interpret the evolutionary and ecological significance of these interactions.

## MATERIALS AND METHODS

### Data collection

All data were collected manually by an extensive search of literature and herbarium specimens, and stored in an Excel spreadsheet (Supplementary Data Table S1). Following the classification established by Těšitel (2016) and Teixeira-Costa and Davis (2021), all ‘endoparasites’ and ‘obligate root parasites’ were included in this survey, whereas ‘euphytoid parasites’, ‘mistletoes’ and ‘parasitic vines’ were excluded. Although endoparasitic mistletoes also exist, some photosynthetic function is often retained in these species and therefore they are excluded here. For each parasite species included, its protologue was checked for mention of a host. Subsequent geographical floras, monographs and peer-reviewed articles in which the species featured were also checked for mention of a host. This broad coverage was achieved using the Royal Botanic Gardens, Kew Library, Art and Archives, in conjunction with online resources, including Biodiversity Heritage Library (biodiversitylibrary.org), JSTOR (jstor.org), GBIF (GBIF.org, 2024) and Index Orobanchaceae (Sánchez Pedraja *et al.*, 2016). Online public observation databases, including iNaturalist (iNaturalist.org) and Plantarium (plantarium.ru), were also consulted, but data were included only when the observation could be verified by an expert.

### Record format

Each report of a host formed the basis of an individual record in the database. A record was not repeated where it was suspected that a host report was simply reiterating a previously published report. However, if the host report was original, then it would be included as a new record even if that host species had previously been reported. For each record, where possible, the following details were documented: the host species, family, order, habit (following Plants of the World Online (https://powo.science.kew.org) classification, e.g. shrub), geographical region (most exact locality of the record as possible), record type (e.g. report in protologue), reference and a confidence rating. We compiled reports from 1061 unique resources, comprising 569 herbarium specimens, 567 literature references and 25 online databases (Supplementary Data Table S5).

### Confidence rating

We acknowledge the significant possibility of errors in the accuracy of these host records. To manage this, we have devised a confidence rating classification. Host records come from a wide range of sources, and each was assessed individually for its accuracy. The classification system is detailed as follows. A rating of ‘high’ was given when: (1) tt was clear that the parasite had been excavated and host contact had been confirmed; (2) the report was made by an expert in this group of plants; (3) a herbarium specimen of the parasite has an identified part of the host attached to it; or (4) the report was on a public observation database and included a photograph where the host connection was clearly visible. A rating of ‘medium’ was given when: (1) it was unclear whether the parasite had been excavated, and it might simply be the nearest plant by observation; (2) the report was published before 1950, unless it is explicitly clear that excavation was performed; (3) a herbarium specimen of the parasite references a host it was collected by but does not specify excavation or include host material on the sheet; or (4) the report was on a public observation database and either included a photograph where the host was visible, or was in the same genus as another host record ranked ‘high’. A rating of ‘low’ was given when: (1) it is made clear the report is dubious by the author; (2) the report is unconfirmed and from a plant family completely unrelated to any other recorded hosts; or (3) the report was on a public observation database that lacked a photograph or was not in the same genus as another host record ranked ‘high’. We complied a total of 2847 records, comprising 94 of low confidence, 2071 of medium confidence and 682 of high confidence (Supplementary Table S5).

### Host diversity and specificity analyses

All analyses were carried out using R v.4.1.2 (R Core Team, 2021). For all analyses, all ‘low’ confidence reports were excluded, to improve the accuracy of the dataset. A parasite species was excluded if it had only one ‘medium’ confidence report. However, this species was maintained if the ‘medium’ confidence report was from a family that featured a high confidence report for at least one other parasite species in the same genus. This step was taken to avoid excluding reports simply on the basis of limited data availability. Only 124 of 2742 records were removed because of this step. These interactions were rechecked manually and confirmed as unlikely given the existing knowledge for each parasite genus or family (low confidence). Thus, this filtering step was retained. With these filtering steps in place, both the ‘high’ and remaining ‘medium’ confidence host reports were considered sufficiently robust for the following analyses.

The dataset was manipulated for analyses using dplyr v.1.1.1 (Wickham *et al.*, 2023) and stringr v.1.5.0 (Wickham, 2023). Host–parasite taxa links and a host diversity heatmap were plotted onto a seed plant phylogeny using ggtree v.3.2.1 (Yu *et al.*, 2017). The family-level seed plant phylogeny used here was obtained from Li *et al.* (2019). The ten host families parasitized by the highest number of genera were plotted in a bar chart using ggplot2 v.3.4.2 (Wickham, 2016). The number of parasitic plants with hosts of each habit type were plotted as a pie chart using ggplot2 v.3.4.2 (Wickham, 2016). Asteraceae hosts were assigned to a tribe following the classification established by Mandel *et al.* (2019). Host specificity at three taxonomic levels (species, genus and family) of each parasite, grouped by parasite family, was plotted as a raincloud plot using ggplot2 v.3.4.2 (Wickham, 2016), see v.0.8.0 (Lüdecke *et al.*, 2023) and gghalves v.0.1.4 (Tiedemann, 2022). The extent of occurrence (in kilometres squared) of each species was calculated using rCAT v.0.1.6 (Moat and Bachman, 2017) from distribution data acquired via mass download from GBIF (https://doi.org/10.15468/dl.hweuk9), and was subsequently cleaned to remove erroneous co-ordinates and verify species names. Counts of holoparasite species, genera and families in different regions of the world are available (Supplementary Data Table S5), filtered to exclude species for which a host record was not recorded in the database. Extent of occurrence is a commonly used metric in conservation studies to determine the approximate geographical range of a species rapidly (IUCN Standards and Petitions Committee, 2022). Although far from accurate, it provides a rough estimation that is sufficient for the needs of this analysis. Extent of occurrence was logarithmically transformed and plotted against host specificity using ggplot2 v.3.4.2 (Wickham, 2016). IUCN rankings were obtained using taxize v.0.9.1 (Chamberlain *et al.*, 2022) to batch search a list of parasite species. Counts of low, medium and high confidence records in each Biodiversity Information Standards Level 2 region (Brummitt, 2001) were calculated and plotted on corresponding shapefiles using ArcGIS Pro v.3.2 (ESRI, 2023). These data are reported (Supplementary Data Table S5). Shapefiles were obtained from the Biodiversity Information Standards (formerly Taxonomic Databases Working Group) github (https://github.com/tdwg/wgsrpd/tree/master).

## RESULTS

**Fig. 1.**
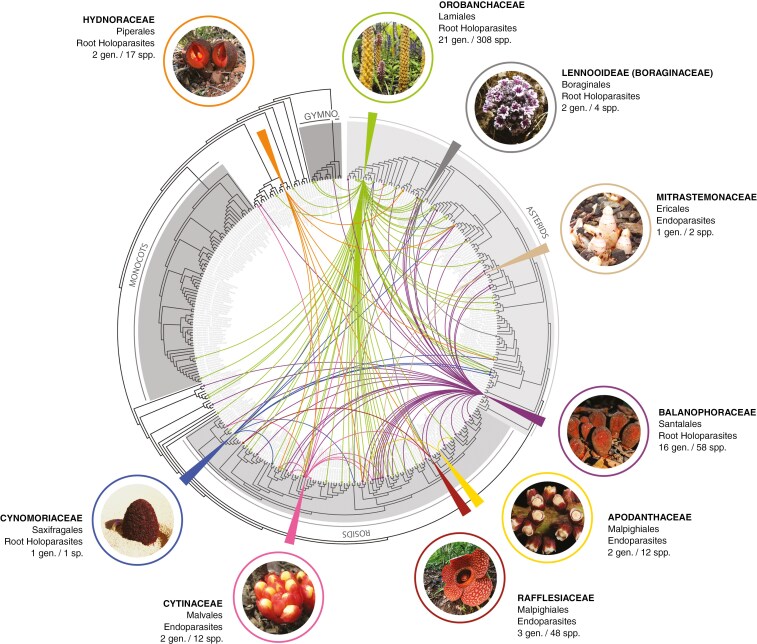
Host–parasite interactions across holoparasitic plants. Arrows represent parasite–host interactions across all nine holoparasite lineages, presented on a family-level seed plant phylogeny (tree from Li *et al.*, 2019), with major clades highlighted and labelled. For each lineage, the following information is provided: family, order, mode of parasitism, the number of parasite genera and species within that lineage, and a photograph of an example species. Photograph credits: Hydnoraceae, Orobanchaceae = Sebastian Hatt; Lennooideae = Joyce Cary; Mitrastemonaceae = Dokudami; Balanophoraceae, Rafflesiaceae, Cytinaceae, Cynomoriaceae = Chris Thorogood; Apodanthaceae = Kevin Thiele.

Holoparasitic plants have evolved independently in nine families of angiosperms, comprising the obligate root parasites [Hydnoraceae, Orobanchaceae, Balanophoraceae and Lennooideae (Boraginaceae)] and endoparasites (Apodanthaceae, Cytinaceae, Mitrastemonaceae and Rafflesiaceae). We report a phylogenetically diverse range of hosts, with records spanning 89 families across 35 orders (Fig. 1; Supplementary Data Table S1). The Orobanchaceae (308 spp.) infect the greatest diversity of host families (62), followed by the Balanophoraceae (58 spp., 42 families).

### Diversity of host preference

**Fig. 2.**
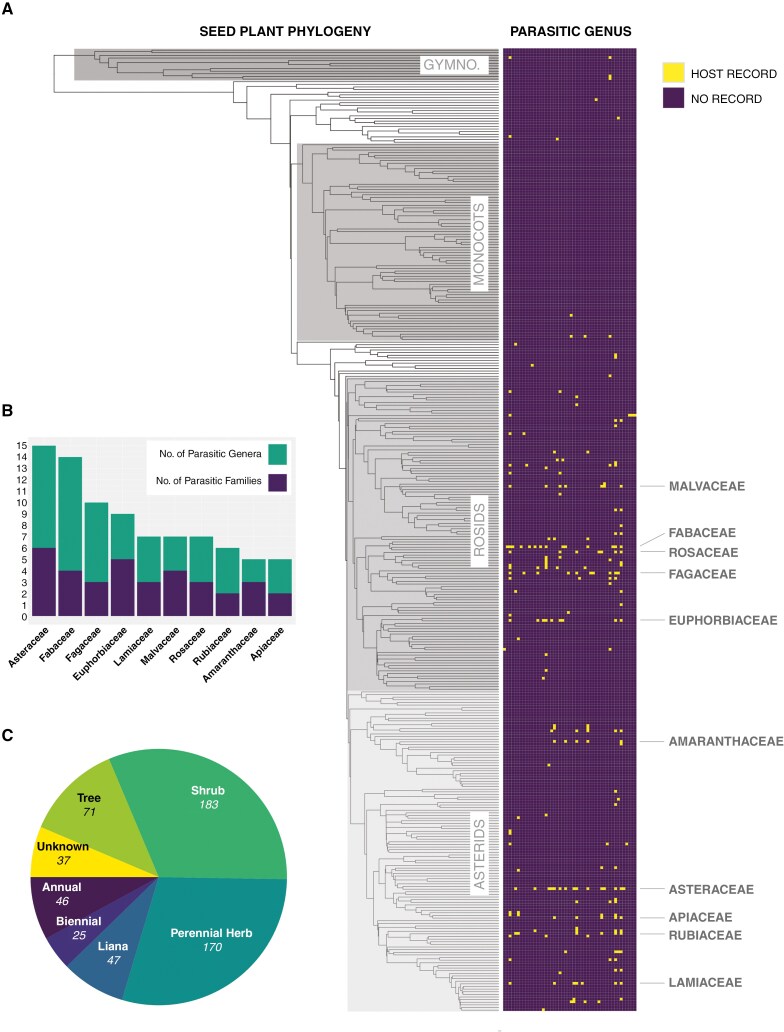
Host diversity of holoparasitic plants. (A) Heat map of host records by parasite genus, applied to a seed plant phylogeny (tree from Li *et al.*, 2019). Heat map squares are coloured yellow if a host family has been recorded for a given parasite genus. The top 10 host families by number of parasitic genera are highlighted on the right. (B) Number of parasitic genera and families for the top 10 host families arranged by number of parasitic genera. (C) The number of parasitic plant species recorded per host habit type.

We acknowledge the uncertainty of many of the host records used in these analyses (see Materials and Methods), particularly in species for which host identification is especially challenging. Notwithstanding, we find the patterns and results presented to be striking and clearly defined. Certain host families are especially prone to parasitism (Fig. 2A; for a fully annotated version, see Supplementary Data Fig. S1). The Asteraceae are the most commonly parasitized, infected by 31.6 % of parasite species (118 of 373), across six of the nine parasite lineages (Supplementary Data Table S2). We examined the distribution of hosts across Asteraceae tribes (Mandel *et al.*, 2019) (Supplementary Data Table S3). We found a strong asymmetry in host diversity, with a surprising near absence of hosts in several globally widespread and species-rich tribes, including the Vernonieae, Mutisieae and Eupatorieae. Furthermore, the largest tribe, the Senecioneae, are parasitized by only 11 parasite species across two families. Other, less species-rich, tribes have considerably more parasites, such as the Inuleae (30 parasite species across six families), Astereae (19/5), Heliantheae (20/4) and Anthemideae (48/3).

The next most commonly parasitized family is the Fabaceae, infected by 16.9 % of parasite species (63 of 373) across four parasite lineages. Some host families show relatively low parasite species counts, but high incidence of convergence across unrelated lineages. For example, Euphorbiaceae hosts are infected by only 16 parasite species, but across five different families. Likewise, species in the genus *Quercus* L. (Fagaceae) are host to only 18 parasite species, but across seven genera in three families. Other host families frequently parasitized by multiple parasite genera and families include Lamiaceae, Malvaceae, Rosaceae, Rubiaceae, Amaranthaceae and Apiaceae (Fig. 2B).

We found a striking absence of hosts in the monocot clade (Fig. 2A), despite comprising 23 % of angiosperm diversity (Christenhusz and Byng, 2016) and containing some of the most species-rich families of angiosperms: the Orchidaceae and Poaceae. Only two of the 77 monocot families are recorded as hosts (Poaceae and Cyperaceae); collectively infected by only 11 parasite species, all belonging to the Orobanchaceae. Gymnosperms are parasitized by only three species. There are no documented hosts in the vascular spore plants (ferns and lycopods).

We assessed the diversity of host habit by grouping host species into annual, biennial or perennial herb, liana, shrub, tree or unknown (where the host was not identified to species level). Approximately 36 % of hosts were shrubs, ~31 % were perennial herbs, and 16 % were trees (Fig. 2C).

### Host specificity across parasite lineages

**Fig. 3.**
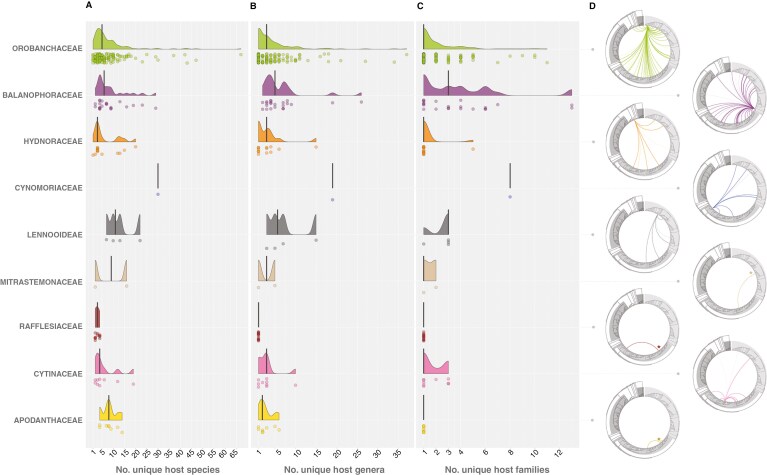
Host specificity of holoparasitic plants. (A–C) The distribution of host specificity across these lineages. Each point represents a parasite species, and the respective curve reflects the density of points across the *x*-axis. Specificity is illustrated at three taxonomic resolutions: number of host species (A), genera (B) and families (C). The data are subset to exclude parasite species with fewer than two observations of a host species, unless that report is high confidence. The median number of host species, genus and family per parasite species is illustrated for each holoparasite lineage by a thick vertical line. (D) For each parasite family, a subset of Fig. 1 is depicted, illustrating the diversity of host–parasite interactions within that family, mapped onto a seed plant phylogeny (Li *et al.*, 2019), with major clades highlighted. For Mitrastemonaceae, Rafflesiaceae and Apodanthaceae, a star indicates the origin of the arrows.

To investigate how host specificity varies between and within parasitic lineages, we plotted the distribution of host species, genus and family counts for each parasite, grouped by lineage (Fig. 3). In the four largest lineages [Orobanchaceae (142), Balanophoraceae (22), Hydnoraceae (13) and Cytinaceae (9)], we observed strongly skewed distributions, each with a small number of outliers (host generalists). To determine the average level of host specificity exhibited per lineage, we calculated the median of each distribution (Fig. 3A). For example, in the Orobanchaceae, a parasite species was found to have an average of five host species, three host genera and one host family. Extreme specialists, those with only a single exclusive host species, are rare, with only eight recorded (excluding species with fewer than two records of the same exclusive host) (Supplementary Data Table S4). Likewise, extreme generalists are uncommon; only 13 species have hosts from six or more families.

**Fig. 4.**
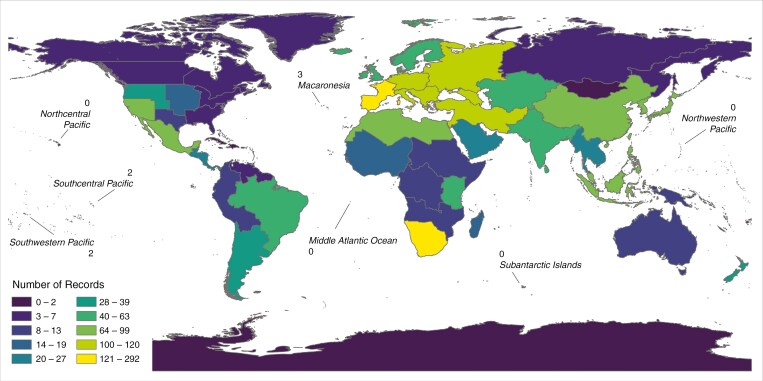
Total number of host records for each geographical region of the world. The total count of host records in the dataset (Supplementary Data Table S1) for each Biodiveristy Information Systems Level 2 region (Brummitt, 2001) of the world is indicated by colour according to the legend. Counts for island regions are indicated directly on the map. This map suggests a potential bias in the dataset against Africa, Australasia, the Pacific and South America.

We found that parasites with few host species and low phylogenetic diversity can still achieve equivalently large geographical ranges (Supplementary Data Fig. S2), demonstrating that large geographical range can be obtained by parasitizing a single (presumably widespread) species, multiple species within a single genus, or multiple families. However, we acknowledge a geographical bias in the dataset, owing to overrepresentation of species from Europe and North America and underrepresentation of species from Africa, Australasia, the Pacific and South America (Fig. 4; Supplementary Data Table S5; Meyer *et al.*, 2016).

## DISCUSSION

### Convergence in host preference

Our results reveal a marked asymmetry in holoparasitic plant host preference for Asteraceae, Fabaceae and Euphorbiaceae. Most striking is the near-complete absence of monocotyledonous hosts, despite composing nearly one-quarter of angiosperm diversity. This points to a common pathway for holoparasitism of eudicot hosts that is shared across the nine independent lineages of holoparasites. Furthermore, this suggests that the developmental pathway to holoparasitism of monocot hosts is interrupted or incompatible for at least one of the critical stages of infection, particularly given that monocots are frequent hosts of hemiparasites.

### Host recognition and seed germination

**Fig. 5.**
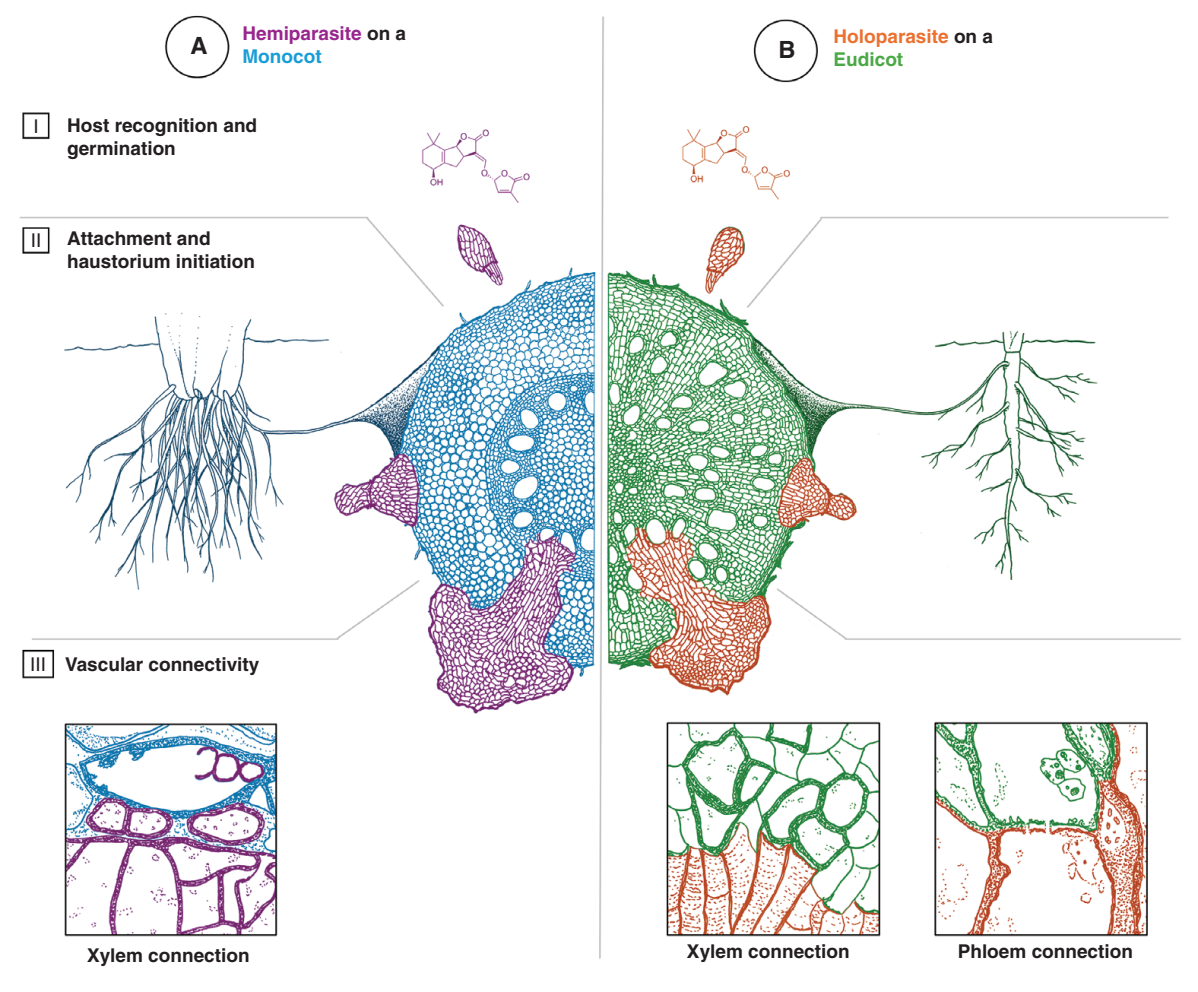
Developmental pathway of a hemiparasite on a monocot host (A) versus a holoparasite on a eudicot host (B). The three stages of parasitism are illustrated in a cross-section of the host root (monocot = blue, eudicot = green) and the germinating parasite seed (hemiparasite = purple, holoparasite = orange). (I) Host recognition and germination. Strigolactones (SLs) released by the host root trigger germination of the parasite seed. No consistent differences have yet been identified between SL diversity in monocots (A) versus eudicots (B), although this is likely to be an artefact of knowledge gaps. (II) Attachment and haustorium initiation. The germinating seed attaches to the host root and develops a haustorium, triggered by host-derived haustorium-inducing factors (HIFs). Monocots (A) have homorhizic root systems, with fine fibrous roots that grow in parallel to the tap root and lack secondary growth. Eudicots (B) have allorhizic root systems, where all roots stem from the primary tap root. Monocot roots are incapable of secondary growth and are often finer than eudicot roots; the comparatively thin roots of a monocot might not be sufficiently reliable to sustain a holoparasite, some of which exhibit slow growth and persist for many years. (III) Vascular connectivity. The parasite haustorium forms a connection with the host vasculature. For both hemiparasites (A) and holoparasites (B), this will involve a direct or indirect connection with the xylem. Most holoparasites (B) will additionally form a direct or indirect connection with the phloem. Illustrated by Sebastian Hatt; (A:III) xylem connection illustration drawn from Masumoto *et al.* (2021): *Striga* parasitizing *Oryza*; (B:III) xylem connection illustration drawn from Tennakoon *et al.* (2007): *Hydnora* parasitizing *Euphorbia*; phloem connection illustration drawn from Krupp *et al.* (2019): *Orobanche* parasitizing *Helianthus*.

Host-elicited germination is the first step in the pathway to parasitism and plays a critical role in determining host specificity (Fig. 5: I) (Xie *et al.*, 2008). Correct host recognition is critical to avoid germination in the presence of a host upon which a parasite cannot establish (suicidal germination) (Huizinga and Bouwmeester, 2023). Many parasitic plants are dependent on perception of host-derived germination stimulants known as strigolactones (SLs) (Xie, 2016); ≥30 unique SLs have been described (Nomura *et al.*, 2024). Asteraceae and Fabaceae are the two families comprising the highest proportion of host species and also contain some of the most well-studied plant species in terms of SL composition. The SLs orobanchol, orobanchyl acetate and 5-deoxystrigol seem to be dominant in both families; indeed, both families exude a similar combination of SLs, potentially suggesting a shared metabolic or regulatory pathway in these two families (Yoneyama *et al.*, 2008, 2010). No consistent differences have been identified between SLs produced by monocots and dicots (Yoneyama *et al.*, 2011), with common SLs, such as sorgomol, 5-deoxystrigol and strigol, recorded from across the angiosperm phylogeny (Wang and Bouwmeester, 2018).

However, current knowledge of SL diversity is very poor, owing, in part, to the practical challenges associated with isolating and identifying SLs (Halouzka *et al.*, 2020). Germination trials of the holoparasite *Hydnora triceps* Drège & E.Mey. (Hydnoraceae) reported germination only in response to root extracts of its exclusive host species and not to closely related, co-occurring hosts (Bolin *et al.*, 2009). Furthermore, *Striga gesnerioides* (Willd.) Vatke, a holoparasite on eudicots, has significantly stricter requirements on SL composition to germinate than *S. hermonthica*, a hemiparasite on monocots (Nomura *et al.*, 2013). The narrow host ranges of most holoparasites suggest that host species possess (as yet undetected) unique combinations of SLs.

### Attachment and haustorium initiation

Successful attachment is influenced by host root architecture (Fig. 5: II). Eudicots are typically allorhizic, comprising a primary tap root from which several orders of lateral roots branch (Osmont *et al.*, 2007). In contrast, monocots are typically homorhizic, whereby multiple adventitious (shoot-borne) roots grow in parallel to the primary tap root (Cheng *et al.*, 2013). When mature, these adventitious roots are often more important for nutrient uptake than the comparatively short-lived tap root (Osmont *et al.*, 2007). Monocot roots are incapable of secondary growth (Petrova *et al.*, 2023) and are often finer than eudicot roots (Bellini *et al.*, 2014). Unlike a eudicot root, which undergoes secondary growth to become woody and enlarge in diameter, the comparatively thin roots of a monocot might not be sufficiently reliable to sustain a holoparasite, some of which exhibit slow growth (Teixeira-Costa, 2021) and persist for many years.

Once attached to the surface of the host root, haustorium development is initiated; a process triggered by host-derived haustorium-inducing factors. At this stage, the host can deploy several methods to block the progress of the parasite (Silva *et al.*, 2002; Cameron *et al.*, 2006; Pérez-de-Luque *et al*., 2006; Yoshida and Shirasu, 2009). However, monocot resistance mechanisms are unlikely to be the cause of the lack of holoparasite infection of monocot hosts; hemiparasites are less dependent on host compatibility and yet many readily parasitize monocots, e.g. *Rhinanthus* (Cameron *et al.*, 2006) or *Triphysaria* Fisch. & C.A.Mey. (Wang *et al.*, 2020).

In frequently parasitized families, such as the Asteraceae or Fabaceae (Fig. 2B), specific features of their root anatomy might facilitate easier access for the haustorium to the host vasculature. For example, the absence of an exodermis appears to be widespread in the Fabaceae (Peterson and Perumalla, 1990; Bramley *et al.*, 2009), which Cameron *et al.* (2005) suggest removes a key physical barrier to the successful establishment of a fully developed haustorium. Other potential physical barriers to parasitism in the root anatomy include lignified tissue, such as cortical sclerenchyma and Casparian strips (Kawa and Brady, 2022). The occurrence and composition of these tissues vary across the angiosperms (Peterson and Emanuel, 1983; Peterson and Perumalla, 1990). In a study of cell wall composition of seven monocots and three eudicots by Schrieber *et al.* (1999), lignin content was higher in the Casparian strips of monocots than dicots. Further research is required to investigate the relationship between host susceptibility and the occurrence of certain tissue types.

### Vascular connectivity

The haustorium develops an invading sinker or penetration peg that eventually reaches the stele and makes contact with the host vascular tissue (Fig. 5: III). This connection can be direct, indirect or both (Teixeira-Costa, 2021). Holoparasites typically require direct xylem–xylem connections, whereas hemiparasites can rely on indirect or direct connections (Spallek *et al.*, 2013). In addition to a xylem connection, a haustorium may develop a phloem connection (Fig. 5: III) (Teixeira-Costa, 2021). There are several cases in which parasite phloem has been detected at the interface, but it has not been possible to confirm phloem–phloem continuity (De Vega *et al.*, 2007; Tennakoon *et al.*, 2007). However, direct symplastic connection between sieve elements has been confirmed in a few species (Dörr and Kollmann, 1995; Krupp *et al.*, 2019). Most reports of phloem connections are from holoparasites, and not one of these cases features a monocot host. One of the few holoparasitic species that infect monocots is *Lathraea purpurea* (Chung *et al.*, 2010). Interestingly, this genus represents one of only two recorded cases of a holoparasite in which the absence of a phloem connection has been confirmed, the other being *Boschniakia* C.A.Mey. ex Bongard (Těšitel, 2016). *Aeginetia* is another genus of holoparasites known to parasitize monocots; although phloem has been detected in the haustorium of *Aeginetia acaulis* (Rajanna *et al.*, 2005), it remains unclear whether a direct connection is made with the host sieve elements.

Monocot roots are typically polyarch, with phloem poles alternating between the xylem, whereas eudicot roots are typically tetrarch, with the xylem arranged in four rays (Sporne, 1974). Eudicot roots will usually undergo secondary growth, with thickening of the root diameter by division of the vascular cambium into secondary xylem and phloem (Strock and Lynch, 2020). Monocot roots lack a vascular cambium, and so do not undergo this process. The absence of phloem connections with monocot hosts might suggest that some aspect of monocot phloem (e.g. the lack of secondary growth) renders them either physiologically incompatible or unable to translocate sugars efficiently enough to meet the demands of the holoparasite. This might contribute to why the hemiparasitic vine *Cuscuta campestris* has been recorded to parasitize grass, but cannot survive with grass as the sole host (Gaertner, 1950; Baráth, 2021).

Although consisting of only 1079 species, Gymnosperms are the dominant component of boreal forests, composing 27 % of the world’s forest cover (Global Forest Resources Assessment, 2020) yet have very few incidences of parasitism. Unlike angiosperms, which use vessel elements for water transport, gymnosperms rely entirely on tracheids, which are less efficient than vessel elements (Roddy, 2019). The lack of vessel elements in gymnosperms might therefore pose an obstacle to efficient host water transport for survival. *Lathraea* (Orobanchaceae) is one of the few parasitic plants able to infect gymnosperms successfully; notably, this genus infects numerous hosts from diverse families. Uniquely for holoparasites, *Lathraea* secretes excess water through hydathodes in its leaf scales (Renaudin and Garrigues, 1967), which might explain how it is able to maintain a water gradient and successfully parasitize gymnosperms.

There are no records of vascular spore plants as hosts for any holoparasites. Unlike flowering plants, which mostly have their vascular tissue arranged in a eustele, spore plants exhibit great diversity of vascular architecture that includes several arrangements of protostele and siphonostele (Suissa and Friedman, 2022). Like Gymnosperms, ferns also have tracheid-based xylem tissue and relatively low water transport capacity (Zhang *et al.*, 2014). Perhaps the combination of weak xylem conductivity and irregular arrangement of vascular tissue might prevent successful holoparasitism of spore plants.

### Nutrient transfer post-attachment

Once a haustorium is developed and a vascular connection achieved, a host must be able to continue to supply adequate nutrients to sustain the parasite. It is possible that certain hosts might be preferred for being rich in valuable nutrients or secondary metabolites, such as reduced nitrogen. It has been suggested that bio-available nitrogen from nitrogen-fixing Fabaceae might enhance growth and flower production in parasitic plants (Gibson and Watkinson, 1991; Seel *et al.*, 1993) and lower rates of herbivory (Adler, 2002). However, there is little evidence to support N-fixation as a significant factor in host quality (Jiang *et al.*, 2008; Scalon and Wright, 2015); indeed, too much nitrogen could even be detrimental to the parasite (Kokla *et al.*, 2022).

Several frequently parasitized host families (e.g. Euphorbiaceae, Asteraceae, Moraceae) contain species that produce latex that is rich in primary and specialised metabolites (Salomé Abarca *et al.*, 2019), although there is currently no research into their potential uptake by parasites. Biochemical interactions at the haustorial interface are complex and remain poorly understood for nearly all holoparasitic plants. Recent research into mycorrhizal and rhizobial associations (Salonen *et al.*, 2000, 2001; Sui *et al.*, 2018) with host–parasite systems suggests that third-party interactions can have significant impact on host quality, and therefore might influence host preference.

Life history presumably plays an important role in determining host suitability. A number of monocot families are aquatic; a niche that appears to be unsuitable for parasitic plants, perhaps owing to the challenges of water-borne host perception and attachment. Likewise, ~70 % of orchids (~18 000 spp.) exist on trees as epiphytes (Gravendeel *et al.*, 2004) and possibly lack sufficient root systems to sustain parasites. Our survey found perennial herbs, shrubs and trees to be the most common hosts, all of which persist for several or more years (Fig. 2C). This allows more time for a parasite to establish and complete its lifestyle than a short-lived host, such as an annual herb. Despite this, 46 holoparasite species were recorded infecting annual herbs. However, only nine of these parasites exclusively parasitize annual herbs. Therefore, this result might simply reflect the knowledge gaps in the dataset, because some of these parasites might also infect perennial hosts, but have not yet been recorded doing so. Further research and detailed field observations of a focus group of parasitic plants (as in the study by Schneeweiss, 2007) would significantly enhance understanding of the relationship between host preference and life form.

The near-complete absence of monocot hosts of holoparasites, despite their substantial representation in angiosperm diversity, suggests the developmental pathway to holoparasitism of monocot hosts is interrupted or incompatible for at least one of the critical four steps. Although there are general biochemical and morphological differences that can be drawn between monocots and eudicots, it is difficult to link these directly to host preference. The key factor driving the absence of monocot hosts of holoparasites might be linked the phloem parasitism, which is a requirement of many holoparasites that has never been recorded on a monocot host. Future research should investigate this by examining the vascular connection in the exceptional cases of holoparasites that infect monocots, such as *Aeginetia* and *Lathraea*.

### Patterns of host specificity: a reflection of unrefined species concepts?

Evolutionary theory suggests there are fitness trade-offs between host specificity and parasite performance (Walker *et al.*, 2017). Generalists benefit from increased availability of potential hosts but suffer from a less intimately adapted relationship with each host, leading to reduced virulence and performance. In contrast, specialists benefit from increased compatibility that enables more efficient nutrient transfer but are restricted by the distribution and availability of a narrower pool of putative hosts. Futhermore, specialism tends to be associated with longer-lived hosts that serve as more predictable resources (Schneeweiss, 2007). Taxonomic rank is an important factor when considering specialism versus generalism. For example, within the parasitic genus *Hydnora* (Hydnoraceae), *Hydnora triceps* is specific to a particular species (*Euphorbia dregeana*), *Hydnora africana* is specific to hosts of a particular genus (*Euphorbia*), and *Hydnora abyssinica* is specific to hosts of a particular family (Fabaceae). Our results suggest that, despite a number of outliers, these fitness trade-offs constrain the majority of parasites to be specific to a small number (up to ~10) host species, often contained within one to few genera, usually within a single family (Fig. 3). Indeed, across all the parasite species included in this study, the median number of host species was five, with three host genera and one host family.

Our results suggest that there are few true single-species specialists: only eight parasite species consistently emerge as single-species specialists (Supplementary Data Table S4). This suggests that parasite compatibility with any single host is rarely intimate enough to exclude even closely related host species. Where such specialism does occur, it may represent a case of niche-partitioning between co-existing species (Schoener, 1974). For example, in Namibia, *Hydnora triceps*, *Hydnora visseri* and *Hydnora africana* frequently co-occur at the same locality, where each species parasitizes a different *Euphorbia* species. Indeed, host-driven divergence has been identified as an important driver of allopatric speciation in *Orobanche minor* (Thorogood *et al.*, 2008).

Our understanding of the host specificity of a parasite is limited by the availability of reliable data, which for many species is very poor (Supplementary Data Fig. S3). Furthermore, the perceived degree of specialism is dependent on the taxonomic circumscription of both the parasite species and its host species. For example, *Orobanche minor* parasitizes ≥67 host species across 11 families, the most of any parasite we examined. However, cryptic races have been identified that are locally adapted to parasitize certain hosts preferentially (Thorogood *et al.*, 2009). Multiple-family generalists, although present in all but two parasite families, also represent a minority of cases (Fig. 3; Supplementary Data Table S4). For example, *Thonningia sanguinea* and *Balanophora fungosa* (Balanophoraceae) jointly parasitize the most host families, at 13 each. However, this might, in part, be a function of poorly collected data; most Balanophoraceae parasitize trees in dense rainforests, where it is almost impossible to confirm the identity of the host, even if the parasite is excavated.

Unlike obligate root parasites, endoparasites are confined within the host tissue (with the exception of flowering and setting seed) and therefore require an intimately adapted relationship with their host in order to ensure the necessary level of host–parasite compatibility for survival. However, our results reveal several endoparasites in the Cytinaceae, Apodanthaceae and Mitrastemonaceae for which a single species might have hosts in multiple genera or even families. A possible explanation is that existing species concepts for these parasites are inadequate. Indeed, there are several reported cases where, seemingly, a generalist parasite species might constitute an assemblage of cryptic species, each with its own distinct preferences (Demanche *et al.*, 2001; McCoy *et al.*, 2001; De Vega *et al.*, 2008; Martinu *et al.*, 2015). The reduced vegetative structures of many holoparasites often obscure classification and identification of species, making it easy to overlook cryptic species even under intense taxonomic scrutiny (Teixeira‐Costa *et al.*, 2021). With this in mind, the multi-modality evident between closely related species in a given lineage, specifically the generalist outliers that strongly contrast against the majority of species with a small host range, suggests that these apparent generalists might constitute several as yet unrecognized cryptic host-specific races. Therefore, we suggest that host specificity data, when available, should be more routinely considered in species concepts for holoparasitic plants. Especially in the conjunction of molecular data, this will help to tease apart morphologically cryptic taxa.

## Conclusion

Our analyses reveal a striking disproportionality in host preference across host lineages, most notably a near-complete absence of monocot hosts despite their substantial contribution to angiosperm diversity. This suggests that the developmental pathway to holoparasitism of monocot hosts is disrupted or incompatible at one or more stages of infection, particularly given that monocots are frequent hosts of hemiparasites. We suggest that this might be linked to the requirement of most holoparasites to establish a phloem–phloem connection, a characteristic not yet observed in haustoria formed with monocot hosts. Furthermore, the unexpected variation in host specificity among closely related species suggests that even apparent generalists might comprise cryptic host-specific taxa, underscoring the need to reconsider parasitic plant species concepts. Our data collectively highlight a complex interplay between ecological and physiological factors shaping the evolution of host–parasite interactions.

## SUPPLEMENTARY DATA

Supplementary data are available at *Annals of Botany* online and consist of the following.

Table S1: database of host preference for all holoparasitic plants. Table S2: the number of host genera and species parasitized within each host family, and the number of parasite families, genera and species that infect each host family. Table S3: the number of host genera and species within each tribe of Asteraceae, and the number of parasite families, genera and species that infect each tribe of Asteraceae. Table S4: the number of unique host families, genera and species for each parasite species (subset to those species with more than two records of at least medium confidence). Table S5: summary statistics for resource types used, records collected, geographical range of parasites, and the proportion of records collected from each geographical region. Figure S1: effect of host specificity on the geographical range of a parasite. Figure S2: availability of reliable host specificity data per parasite family. Figure S3: a fully annotated version of Fig. 2A.

mcae180_suppl_Supplementary_Table_S1

mcae180_suppl_Supplementary_Tables_S4-S5

mcae180_suppl_Supplementary_Figure_S3

mcae180_suppl_Supplementary_Table_S2

mcae180_suppl_Supplementary_Table_S3

mcae180_suppl_Supplementary_Figure_S1

mcae180_suppl_Supplementary_Figure_S2
