# Supplementary material for: Parasitic plants show striking convergence in host preference across angiosperm lineages
Source: Ann Bot. 2025 Jul 14;135(6):1135–46. doi: 10.1093/aob/mcae180 (PMC12259541; doi:10.1093/aob/mcae180)
Supplement: mcae180_suppl_Supplementary_Figure_S3 [file mcae180_suppl_supplementary_figure_s3.pptx]

## Slide 1
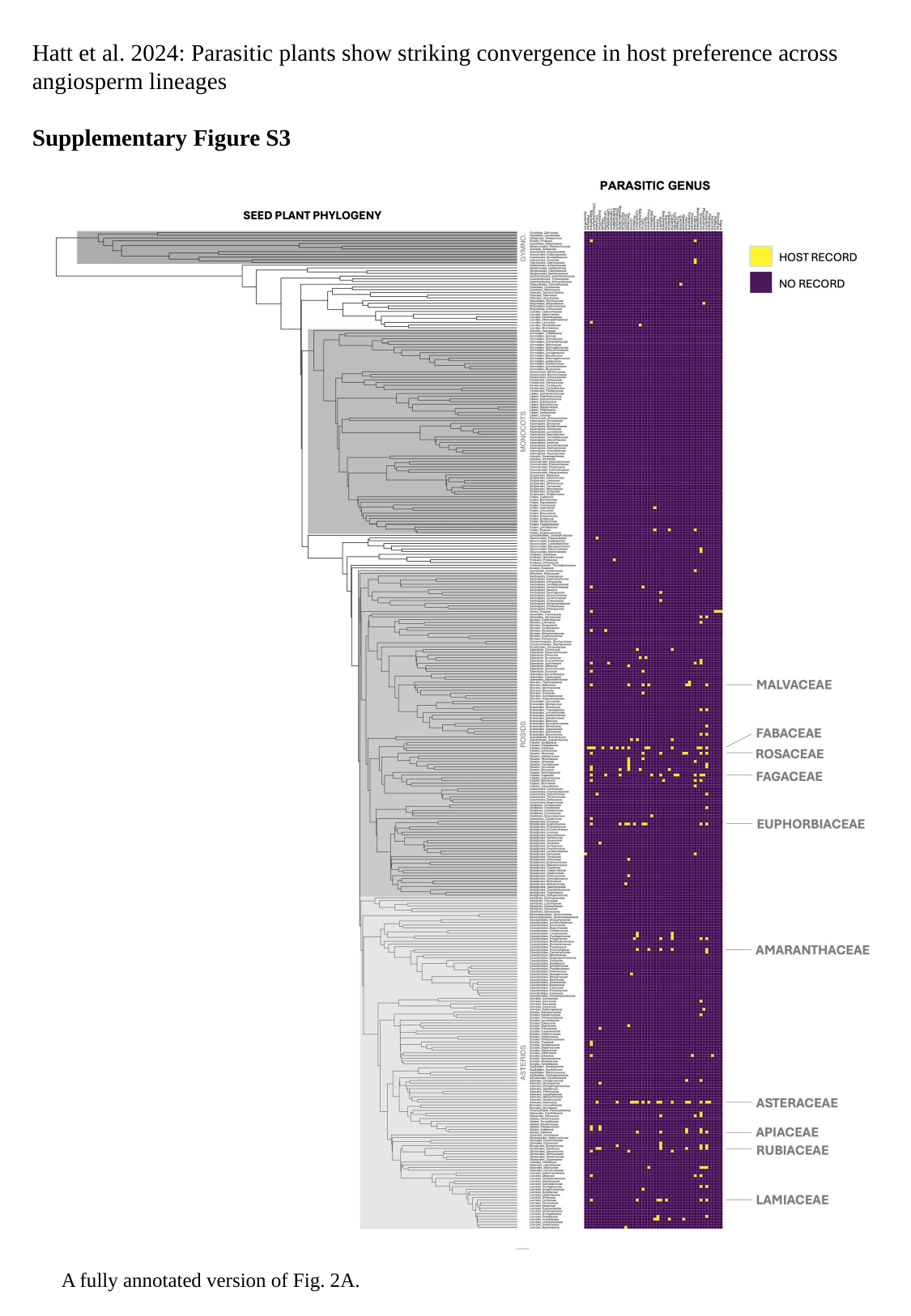

Hatt et al. 2024: Parasitic plants show striking convergence in host preference across angiosperm lineages
Supplementary Figure S3
A fully annotated version of Fig. 2A.
