# Supplementary material for: Parasitic plants show striking convergence in host preference across angiosperm lineages
Source: Ann Bot. 2025 Jul 14;135(6):1135–46. doi: 10.1093/aob/mcae180 (PMC12259541; doi:10.1093/aob/mcae180)
Supplement: mcae180_suppl_Supplementary_Figure_S1 [file mcae180_suppl_supplementary_figure_s1.pptx]

## Slide 1
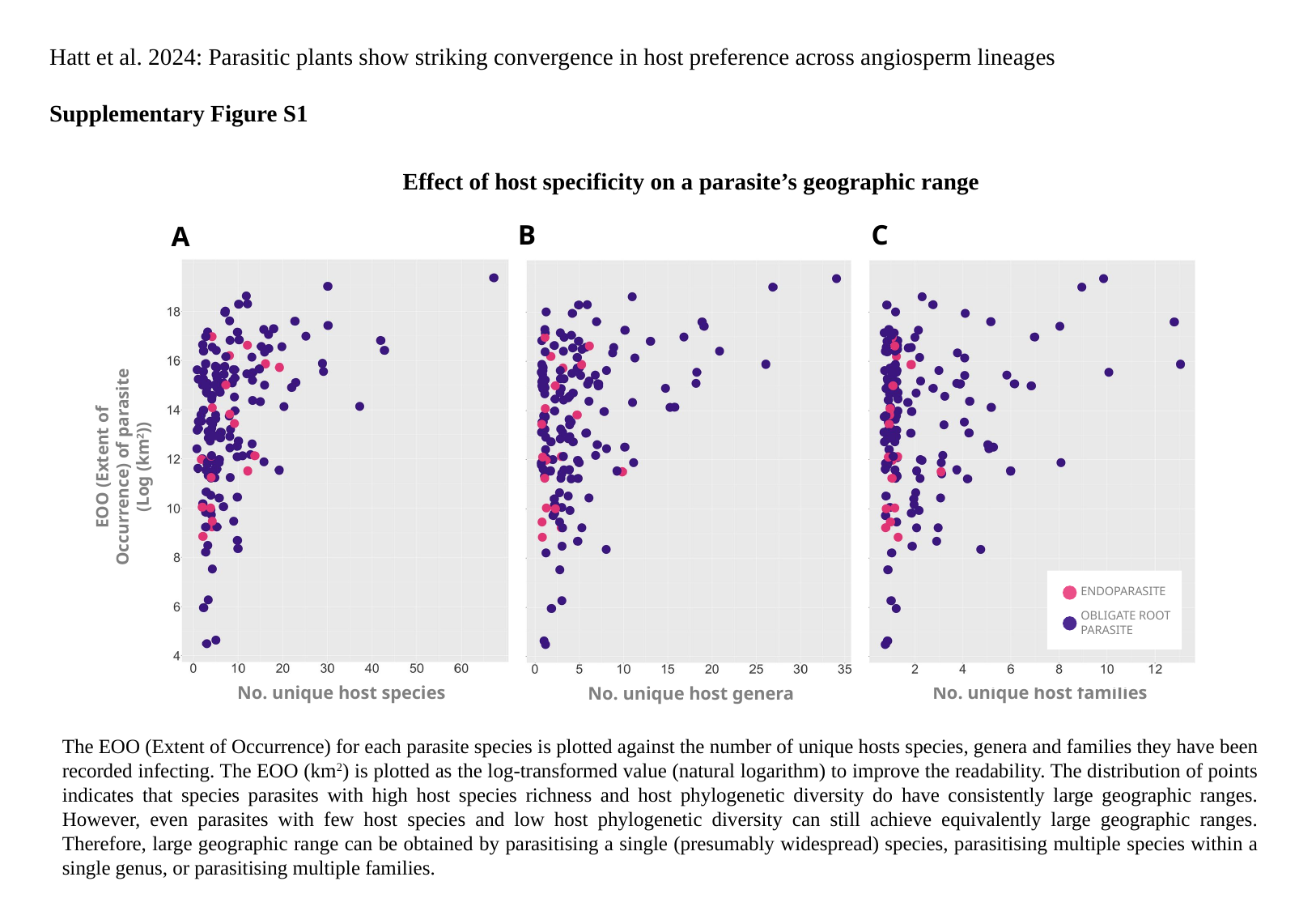

Hatt et al. 2024: Parasitic plants show striking convergence in host preference across angiosperm lineages
Supplementary Figure S1
Effect of host specificity on a parasite’s geographic range
B
C
A
EOO (Extent of Occurrence) of parasite (Log (km2))
ENDOPARASITE
OBLIGATE ROOT PARASITE
No. unique host families
No. unique host species
No. unique host genera
The EOO (Extent of Occurrence) for each parasite species is plotted against the number of unique hosts species, genera and families they have been recorded infecting. The EOO (km2) is plotted as the log-transformed value (natural logarithm) to improve the readability. The distribution of points indicates that species parasites with high host species richness and host phylogenetic diversity do have consistently large geographic ranges. However, even parasites with few host species and low host phylogenetic diversity can still achieve equivalently large geographic ranges. Therefore, large geographic range can be obtained by parasitising a single (presumably widespread) species, parasitising multiple species within a single genus, or parasitising multiple families.
