# Supplementary material for: Parasitic plants show striking convergence in host preference across angiosperm lineages
Source: Ann Bot. 2025 Jul 14;135(6):1135–46. doi: 10.1093/aob/mcae180 (PMC12259541; doi:10.1093/aob/mcae180)
Supplement: mcae180_suppl_Supplementary_Figure_S2 [file mcae180_suppl_supplementary_figure_s2.pptx]

## Slide 1
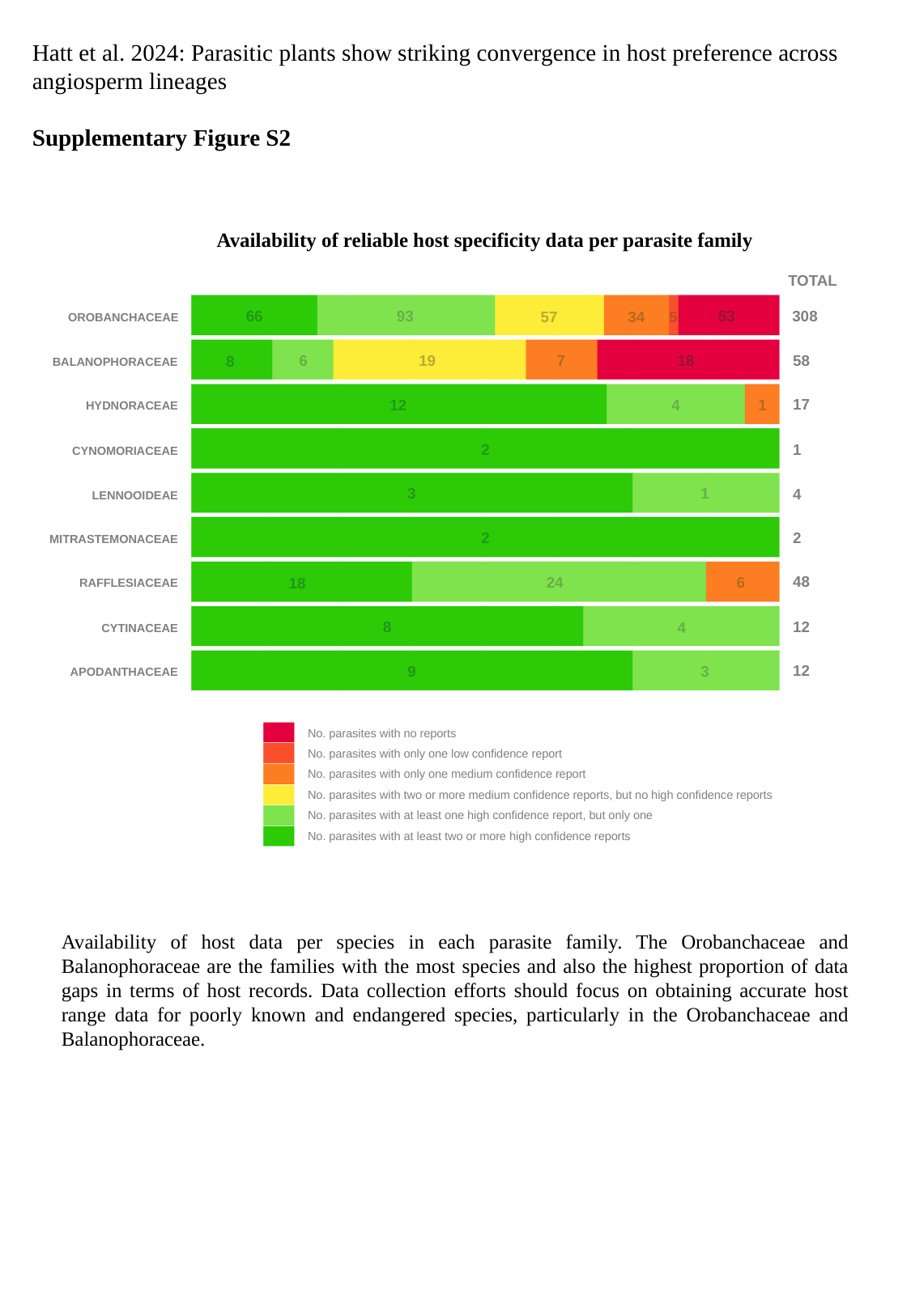

Hatt et al. 2024: Parasitic plants show striking convergence in host preference across angiosperm lineages
Supplementary Figure S2
Availability of reliable host specificity data per parasite family
TOTAL
53
93
66
308
5
34
57
OROBANCHACEAE
18
6
19
7
58
8
BALANOPHORACEAE
17
4
1
12
HYDNORACEAE
1
2
CYNOMORIACEAE
1
3
4
LENNOOIDEAE
2
2
MITRASTEMONACEAE
48
6
24
18
RAFFLESIACEAE
8
12
4
CYTINACEAE
12
3
9
APODANTHACEAE
No. parasites with no reports
No. parasites with only one low confidence report
No. parasites with only one medium confidence report
No. parasites with two or more medium confidence reports, but no high confidence reports
No. parasites with at least one high confidence report, but only one
No. parasites with at least two or more high confidence reports
Availability of host data per species in each parasite family. The Orobanchaceae and Balanophoraceae are the families with the most species and also the highest proportion of data gaps in terms of host records. Data collection efforts should focus on obtaining accurate host range data for poorly known and endangered species, particularly in the Orobanchaceae and Balanophoraceae.
